# Screening the elite chemotypes of *Gloriosa superba* L. in India for the production of anticancer colchicine: simultaneous microwave-assisted extraction and HPTLC studies

**DOI:** 10.1186/s12870-021-02843-8

**Published:** 2021-02-05

**Authors:** Devendra Kumar Pandey, Prabhjot Kaur, Vijay Kumar, R. M. Banik, Tabarak Malik, Abhijit Dey

**Affiliations:** 1 Department of Biotechnology, School of Biotechnology and Biosciences, Lovely Professional University, Phagwara, Punjab 144411 India; 2 School of Biochemical Engineering, Indian Institute of Technology BHU, Varanasi, India; 3 Department of Biochemistry, College of Medicine and Health Sciences, University of Gondar, Gondar, Ethiopia; 4 Department of Life Sciences, Presidency University, 86/1 College Street, Kolkata, 700073 India

**Keywords:** Altitudinal variation, Chemotypes, Colchicine, HPTLC, Microwave assisted extraction, Response surface methodology

## Abstract

**Background:**

*Gloriosa superba* L. (Colchicaceae) is a high-value medicinal plant indigenous to Africa and Southeast Asia. Its therapeutic benefits are well-established in traditional medicines including Ayurveda. It is well known for its natural bioactive compound colchicine which exhibits a wide range of pharmacological activities i.e. rheumatism, gout and was also introduced into clinical practices. The increasing demand as well as its illegal harvesting has brought this valuable plant under threatened category.

**Methods:**

The present investigation describes a microwave assisted extraction (MAE) strategy coupled with a densitometric-high performance thin layer chromatographic (HPTLC) methodology for the analysis of colchicine from 32 different populations of *G. superba*. A Box-Behnken statistical design (3 level factor) has been employed to optimize MAE, in which power of microwave, time of irradiation, aqueous ethanol and pH were used as independent variables whereas colchicine was used as the dependent variables. Chromatography was carried out on Silica gel 60 F_254_ TLC plates with toluene: methanol, 85:15 (v/v) being used as solvent system. Densitometric measurement was performed at λ=254 nm following post-derivatization (10% methanolic sulphuric acid).

**Results:**

Optimal conditions for extraction to obtain the maximum colchicine yield was found to be 7.51 mg g^− 1^ which was very close to be predicted response 7.48 mg g^− 1^ by maintaining microwave power (460 W), irradiation time (6.4 min), aqueous ethanol-30, pH -3. Colchicine content ranged between 2.12–7.58 mg g^− 1^ among 32 *G. superba* populations in which only three chemotypes viz. GS- 1, GS- 3, and GS- 2 collected from West Bengal and Sikkim, respectively exhibited maximum yield of colchicine.

**Conclusion:**

Therefore, this newly developed optimized MAE coupled with HPTLC densitometry methodology not only quantifies colchicine in order to identify elite chemotypes of *G. superba*, but it also encourages in selecting high yielding populations of the plants for industrial use and economic boost for the farmers. This validated, simple and reproducible HPTLC protocol is being used for the first time to estimate colchicine from natural populations of *G. superba* obtained from 32 different geographical regions of India.

**Graphical abstract:**

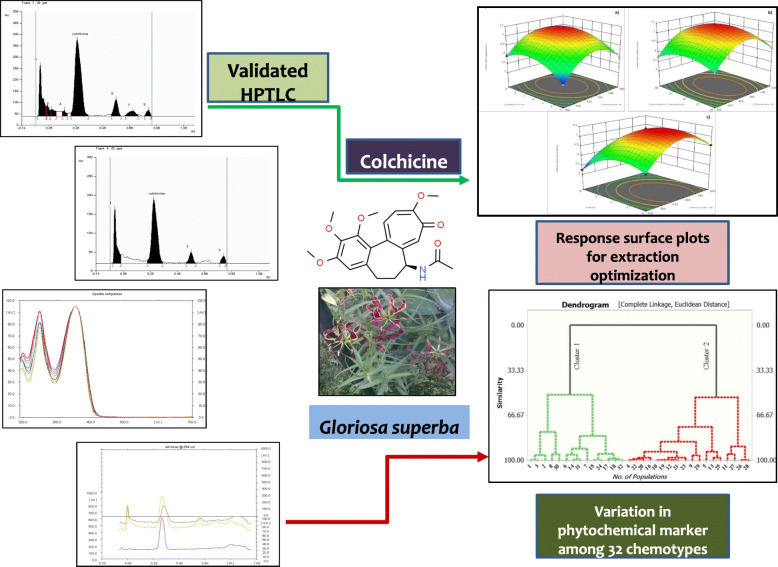

## Background

**Fig. 1:**
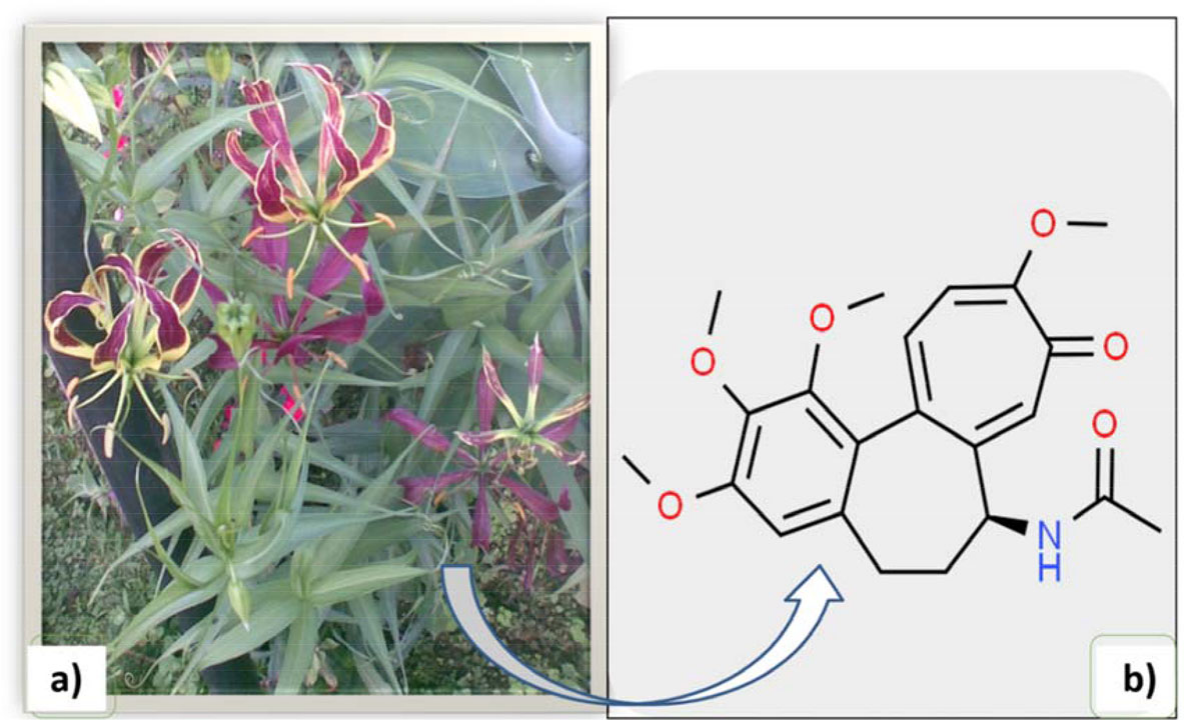
**a** Habit of *G. superba*; **b** Structure of colchicine (from www.chemspider.com)

*Gloriosa superba* L. (Family: Colchicaceae) (Fig. 1a) is herbaceous perennial semi-woody climber [1] native of tropical Asia and Africa. It is found growing all over tropical India at an altitude of 2120 meters from the North West Himalaya to Assam and the Deccan peninsula. In Karnataka, it is generally found growing all along the Western Ghats; it is also found growing in Madagascar, Sri Lanka, Indo-China and in the adjacent islands [2–4]. The genus derives its name from the Latin word gloriosus, referring to the flower. *G. superba* is the national flower of Zimbabwe and also the State flower of Tamil Nadu. *G. superba* is considered as cash crop like sugarcane and cotton due to its high returns. In 2019, the average net return per acre of the *G. superba* cultivation was reported about Rs. 1,499,002 per acre per crop [5].

It is also known by its trade name ‘Glory lily’; in English it is known as ‘Malabar glory lily’ and, in Hindi and Sanskrit as ‘Kalihari’ and ‘Agnisikha’, respectively [6–8]. *G. superba* is a commercially imperative medicinal plant with wide range of biological activities. It is one of the prestigious herbs in Yunani system of medicine. In tropical Africa and Asia, folkloric use of the plant includes all of its parts and indigenous people exploit its value as barter from the traders [9–11]. It’s most popular and widespread use in traditional medicine is manifested against rheumatism and gout [12]. The tubers of the plant are traditionally used to treat chronic ulcers, colic, bruises and sprains, haemorrhoids, leprosy, cancer, and also as a labour pain inducer [13–16]. Pharmacological properties of the tuber indicate its use as an abortifacient, as well as anthelmintic, tonic and stomachic in smaller doses. The leaves are employed to treat piles, ulcers and to expel the placenta, whereas the seeds are used to cure medical conditions in relation to cancer [17].

The medicinal value of the Glory lily is particularly due to the alkaloids especially colchicine, thiocolchicine and gloriosine as well as to the presence of 10 non-alkaloidal compounds *viz*. β-sitosterol, stigmasterol, chelidonic acid, luteolin, etc. [12, 18, 19]. Colchicine is used as a mitosis-arresting agent and is used in cancer treatment and diabetics in addition to promoting polyploidy in agricultural crops.

Colchicine (Fig. 1b) is a major alkaloid of *G. superba* tuber which has shown multifaceted pharmacological properties that has resurfaced since it acts at the subcellular level. Colchicine was introduced into clinical practice more than 200 years ago and is still used as an effective drug to treat gout, a common ailment in the temperate parts of the world [20]. United States Food and Drug Administration (FDA) (2009) have approved the use of colchicine in the treatment of gout. This compound is also widely utilized in the inhibition of polymerization of microtubules and inhibiting fibrosis in experimental animal models [21, 22]. It is also used to treat cancer [23, 24], rheumatism and cardiovascular diseases [25–27]. Colchicine is found in significant quantities only in two plants *viz*. *Colchicum autumnale* and *Gloriosa* sp. [28]. In *G. superba,* colchicine yield ranges from 0.15% - 0.3% in the rhizomes, and 0.7% - 0.9% in the seeds [5]. The discovery of high colchicine content in this plant increased its demand in domestic and worldwide markets.

Due to the over-exploitation and indiscriminate collection and the constraints faced in field cultivation, Glory lily plant is on the verge of extinction from the wild. Therefore, it has been cited as a threatened species in the Red Data Book (2001) by the International Union for Conservation of Nature and Natural Resources (IUCN) [1, 29]. Hence, to ensure its continuous supply and to meet the ever-increasing demand of the herbal medicine industry, screening of elite germplasms with high colchicine content and alternative approaches aiming for enhanced production of colchicine from this valuable medicinal plant are main priorities to the researchers. The identification of elite germplasms will support the use of good quality botanicals suitable for commercial cultivation of *G. superba*. Few reports are available on the exploration of elite chemotypes from different geographical regions of India *viz*. Sikkim and Gangetic plain of India [30–32]. However, very little information is available on the determination of colchicine in *G. superba* using HPTLC-densitometric analysis [31–33] which has been reported as a simple, fast and cost-effective method for quantitative estimation of valuable phytochemicals [34–37].

Furthermore, quality control of crude drugs is a basic requirement for the industry and allied organizations dealing with herbal formulations or natural products. In addition, optimization and development of chromatographic profiles based on marker compounds to assist the appropriate drug production is of utmost importance. Considering the constraints of conventional extraction processes (time, energy and solvent consumption), special attention is required to optimize green and sustainable extraction methods [38]. Optimized green extraction methods produce safe and high-quality bio-active compounds in minutes with high reproducibility and low solvent consumption. In the last two decades, microwave assisted extraction (MAE) has been successfully employed as “green extraction method” for the recovery of industrially important compounds from plant matrices [39–41]. For optimization of the linear and interactive effects of different response variables, response surface methodology (RSM) is an efficacious statistical as well as mathematical design to get maximum production of value-added bioactive compounds [42–44].

Therefore, the aims of the current study are: 1) to optimize the significant variables of microwave-assisted extraction (MAE) employing RSM; 2) To analyse the intrinsic intra-specific disparity in the colchicine amount in the 32 *G. superba* populations collected from 32 different districts across 11 states of India using a rapid, cost-effective and validated HPTLC method; and 3) To screen the elite (high-yielding) germplasms of *G. superba* in order to verify and validate the quality and authenticity of the plant material in terms of colchicine content collected from different geographic locations to fulfil the ever-expanding needs of the medicinal plant markets and the pharmaceutical industries.

## Results

### Plackett-Burman design

**Fig. 2:**
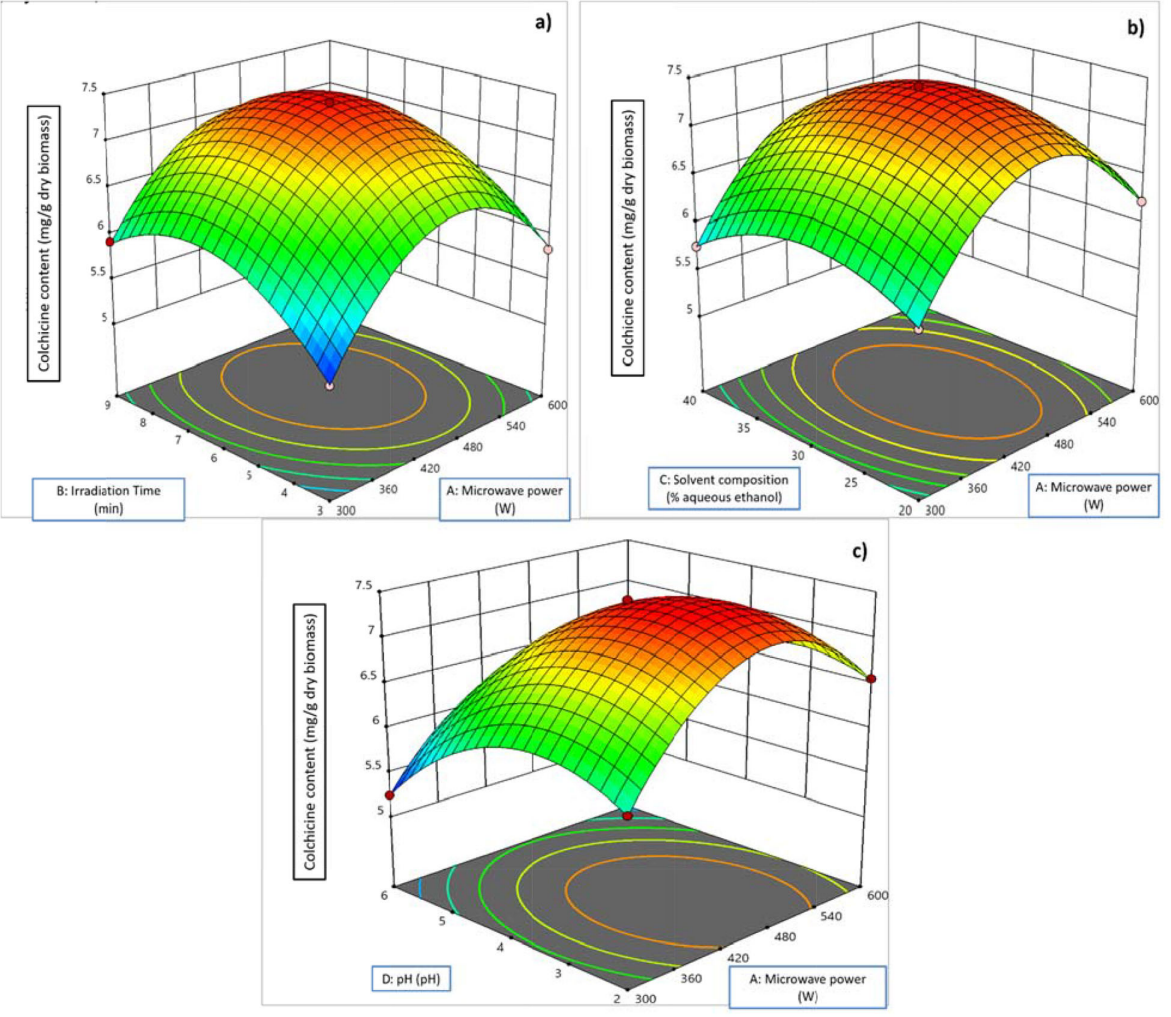
Response surface plots showing interaction between microwave power and irradiation time (**a**); microwave power and solvent composition (**b**); microwave power and pH (**c**)

Plackett-Burman design was employed for the screening of important extraction variables in order to analyse the influence of seven independent variables on the colchicine yield (Table 1). Here, the chosen designed matrix and the resulting colchicine yield from *G. superba* tuber is presented in Table 1. The influence of the parameters of extraction on the extraction of colchicine was evaluated by the methods of regression analysis (Table 2). In this, only four variables such as power of microwave, time of irradiation, solvent composition and pH had exhibited noteworthy influence on colchicine yield as recorded by their *P* values at the level of 5% (*P* < 0.05 values depicted in Table 3). In the present study, colchicine yield as accomplished using Plackett-Burman design has reported disparity up to 2.82-6.66 mg/g dry wt. (Fig. 2).

**Table 1:** Plackett-Burman design and analysis of variance (ANOVA) for experimental results for extraction of colchicine

Std. order	Microwave power (Watt)	Irradiation time (min)	Particle size (mm)	Solvent composition (%aqueous)	Solid: solvent	pH	Extraction steps	Colchicine (mg/g dry wt.)	Predicted
1	600	3	1	20	15	2	2	6.104	6.11
2	600	9	0.5	40	15	2	1	4.980	5.01
3	300	9	1	20	30	2	1	6.660	6.488
4	600	3	1	40	15	6	1	2.824	2.88
5	600	9	0.5	40	30	2	2	5.041	5.007
6	600	9	1	20	30	6	1	5.304	5.476
7	300	9	1	40	15	6	2	2.824	2.492
8	300	3	1	40	30	2	2	3.624	3.885
9	300	3	0.5	40	30	6	1	1.864	1.877
10	600	3	0.5	20	30	6	2	4.344	4.102
11	300	9	0.5	20	15	6	2	3.390	3.721
12	300	3	0.5	20	15	2	1	5.224	5.120

**Table 2:** Response Surface Regression (coded units) and Analysis of Variance (ANOVA) for colchicine compound

Term	Coef	SE Coef	Sum of Squares	dF	Mean Square	F-value	***p***-value
**Constant**	7.37333	0.01560	9.3700	14	0.6695	917.01	< 0.0001^*^
A	0.19092	0.00780	0.4374	1	0.4374	599.06	< 0.0001^*^
B	0.20350	0.00780	0.4969	1	0.4969	680.63	< 0.0001^*^
C	−0.04550	0.00780	0.0248	1	0.0248	34.03	< 0.0001^*^
D	−0.46208	0.00780	2.5600	1	2.5600	3509.30	< 0.0001^*^
AB	−0.05875	0.01351	0.0138	1	0.0138	18.91	0.0009^*^
AC	0.00500	0.01351	0.0001	1	0.0001	0.13	0.7178
AD	−0.09100	0.01351	0.0331	1	0.0331	45.37	< 0.0001^*^
BC	0.01375	0.01351	0.0008	1	0.0008	1.04	0.3289
BD	0.16700	0.01351	0.1116	1	0.1116	152.79	< 0.0001^*^
CD	−0.07775	0.01351	0.0242	1	0.0242	33.12	< 0.0001^*^
A^2^	−0.95550	0.01170	4.8700	1	4.8700	6668.97	< 0.0001^*^
B^2^	−0.59338	0.01170	1.8800	1	1.8800	2571.91	< 0.0001^*^
C^2^	−0.41538	0.01170	0.9202	1	0.9202	1260.31	< 0.0001^*^
D^2^	−0.61900	0.01170	2.0400	1	2.0400	2798.84	< 0.0001^*^
Residual			0.0088	12	0.0007		
Lack of Fit			0.0063	10	0.0006	0.5104	0.8086
Pure Error			0.0025	2	0.0012		
Cor Total			9.3800	26			

A-Microwave power; B-Irradiation Time; C-Solvent composition; D-pH

*Coef* Regression Coefficients, *SE Coef* Standard Error of Regression Coefficient, *Df* Degree of Freedom

*Very significant, where *p* < 0.01

**Table 3:** Box Behnken Design (BBD) with four response variables: Microwave power (A), Irradiation Time (B), Solvent composition (C) and pH (D)

Run	Response variables				Colchicine content (mg/g) dry biomass	
	Microwave power (W)	Irradiation Time (min)	Solvent composition (% aqueous ethanol)	pH		
					Experimental	Predicted
1	300	9	30	4	5.920	5.703
2	450	6	30	4	7.370	7.037
3	450	6	30	4	7.410	6.865
4	450	6	40	6	5.732	6.272
5	600	3	30	4	5.836	5.953
6	450	3	30	2	6.583	6.818
7	300	6	30	2	5.980	5.486
8	600	6	40	4	6.156	6.534
9	450	9	30	6	6.063	5.917
10	600	6	20	4	6.230	6.448
11	450	6	20	2	6.780	6.401
12	300	6	20	4	5.849	5.919
13	450	9	30	2	6.624	6.340
14	300	6	40	4	5.755	6.346
15	600	9	30	4	6.159	5.969
16	600	6	30	2	6.560	6.540
17	600	6	30	6	5.456	5.917
18	300	6	30	6	5.240	5.622
19	450	3	40	4	6.110	5.729
20	450	6	30	4	7.340	6.557
21	300	3	30	4	5.362	5.598
22	450	6	40	2	6.843	6.654
23	450	3	20	4	6.234	5.917
24	450	3	30	6	5.354	5.831
25	450	6	20	6	5.980	6.861
26	450	9	20	4	6.612	6.248
27	450	9	40	4	6.543	6.586

#### Box-Behnken design (BBD) model fitting

RSM is a systematic and contemporary statistical device employed in bioprocess optimization and modelling strategies where diverse design sets are used for problem analysis. RSM is composed of different mathematical designs of which BBD has been applied successfully to attain the enhanced values for extraction in diverse medicinal and aromatic botanicals. Response variables possess linear, quadratic and interaction relations to elicit better yield of the coveted bioactive pharmaceuticals during the extraction process.

Hence, to confirm the influenced extraction variables as well as to calculate the response’s internal variability. BBD is employed for computational analysis of the relations exist among diverse response variables consisting of linear, quadratic as well as 2-way interaction model based on the Eq. 1 as mentioned below. Analyses for multiple regression were carried out to produce the predicted response values (mg/g dry wt.) for colchicine yield (Y).

1 $$ {\displaystyle \begin{array}{c}\mathrm{Y}=7.37+0.1909\mathrm{A}+0.2035\mathrm{B}-0.0455\mathrm{C}-0.4621\mathrm{D}-0.0588\mathrm{AB}+0.0050\mathrm{AC}-0.0910\mathrm{AD}+\\ {}0.0137\mathrm{BC}+0.1670\mathrm{BD}-0.0777\mathrm{CD}-0.9555{\mathrm{A}}^2-0.5934{\mathrm{B}}^2-0.4154{\mathrm{C}}^2-0.6190{\mathrm{D}}^2\end{array}} $$

**Fig. 3:**
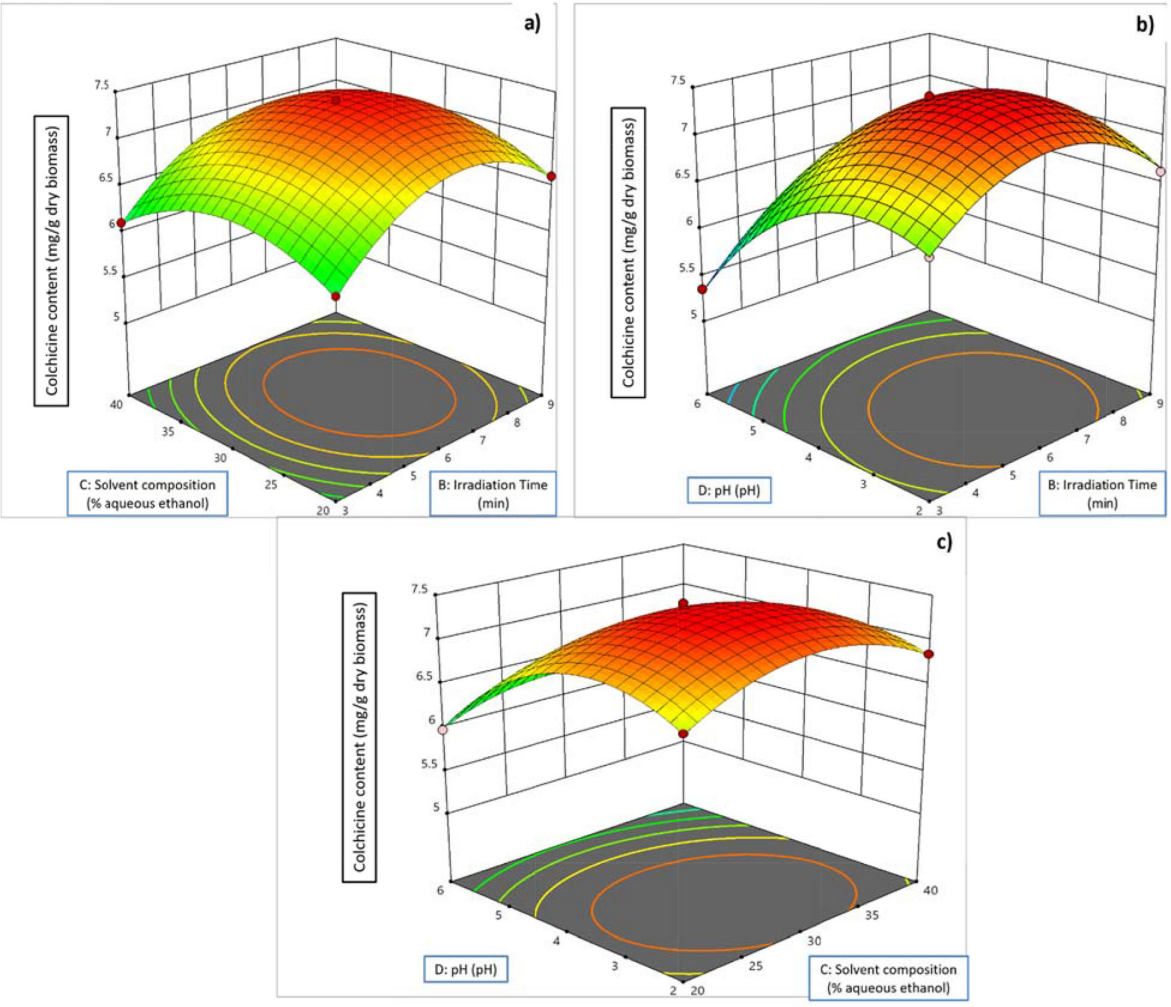
Response surface plots showing the effects of interaction between irradiation time and solvent composition (**a**); irradiation time and pH (**b**); solvent composition and pH (**c**)

[A, B, C, D: power of microwave (A: 300 to 600 W), irradiation time (B: 3-9 minutes), solvent-composition (C: 20%-40% aqueous ethanol) and pH (2-6) respectively which were employed as four MAE independent variables in order to achieve enhanced colchicine levels from *G.superba*. Experimental values of content (%) of colchicine were evaluated by HPTLC methods. Table 3 exhibits the BBD along with the three different independent variables at three different levels and the experimentations besides the anticipated value acquired from colchicine yield. The predicted values obtained from colchicine were correlated with the actual experimentation values. The values for colchicine ranged from 3.445 mg g^-1^ to 7.445 mg g^-1^. Response surface plots showing interaction between different independent variables are presented in Figs. 2 and 3.

#### Fitting the model

Analysis of variance (ANOVA) was calculated for the fitness probability based upon quadratic equations of second-order (Eqs. 1 and 2). ANOVA scores recorded for linear, quadratic as well as interaction for independent variables are exhibited in Table 2.

The probability (*P*) value was assumed highly significant that exhibits lesser than 0.01. Values lesser than 0.05 were noted significant and were reckoned in quadratic equation. *P* values greater than 0.05 were not considered in the quadratic equation as they were non-significant.

The usefulness of the model in explaining all the variations was indicated by the determination coefficients (R^2^) of 99.9 respectively. It was assumed that the proximity of the quality of R^2^ to 1 indicates superior fitment of the model of experimentation to the actual data. On the contrary, lesser the R^2^ value, the slighter relation the dependent variables in the model possess in order to explain the independent variables’ behaviour.

For colchicine yield, the linear effect of the power of microwave (A) was reported to be statistically (*P*≤ 0.00) noteworthy (Table 3). The negative quadratic influence of power of microwave (A^2^) complied with the extraction yields’ deceleration. Considering the time of extraction (B), there was a positive linear effect, which confirms that the compound yield is directly proportional to the increase in time. The negative quadratic influence of the time of extraction (B^2^) proves the reduction in the extraction yield following the Fick’s second law of diffusion. With relation to the solid-solvent ratio (C), all responses exhibit the positive linear as well as negative quadratic effects. The interactive relations between microwave power * irradiation time, irradiation time * pH, irradiation time * aqueous ethanol, irradiation time * pH and aqueous ethanol * pH for colchicine yield were significant. The graph exhibiting the main influence of the independent variables for colchicine yield are presented in Figs. 2 and 3.

As shown in Figs. 2 and 3, the optimized conditions for extraction using microwave i.e., microwave power-irradiation time-aqueous ethanol, pH for the better yield of colchicine were found to be irradiation time (6.4 min), microwave power (460 W), aqueous ethanol (30%), pH (3). At these optimized parameters for microwave extraction, maximum predicted yield achieved for colchicine was found to be 7.51 mg g^-1^ which was very close to be predicted response 7.48 mg g^-1^ respectively.

### HPTLC analysis

**Fig. 4:**
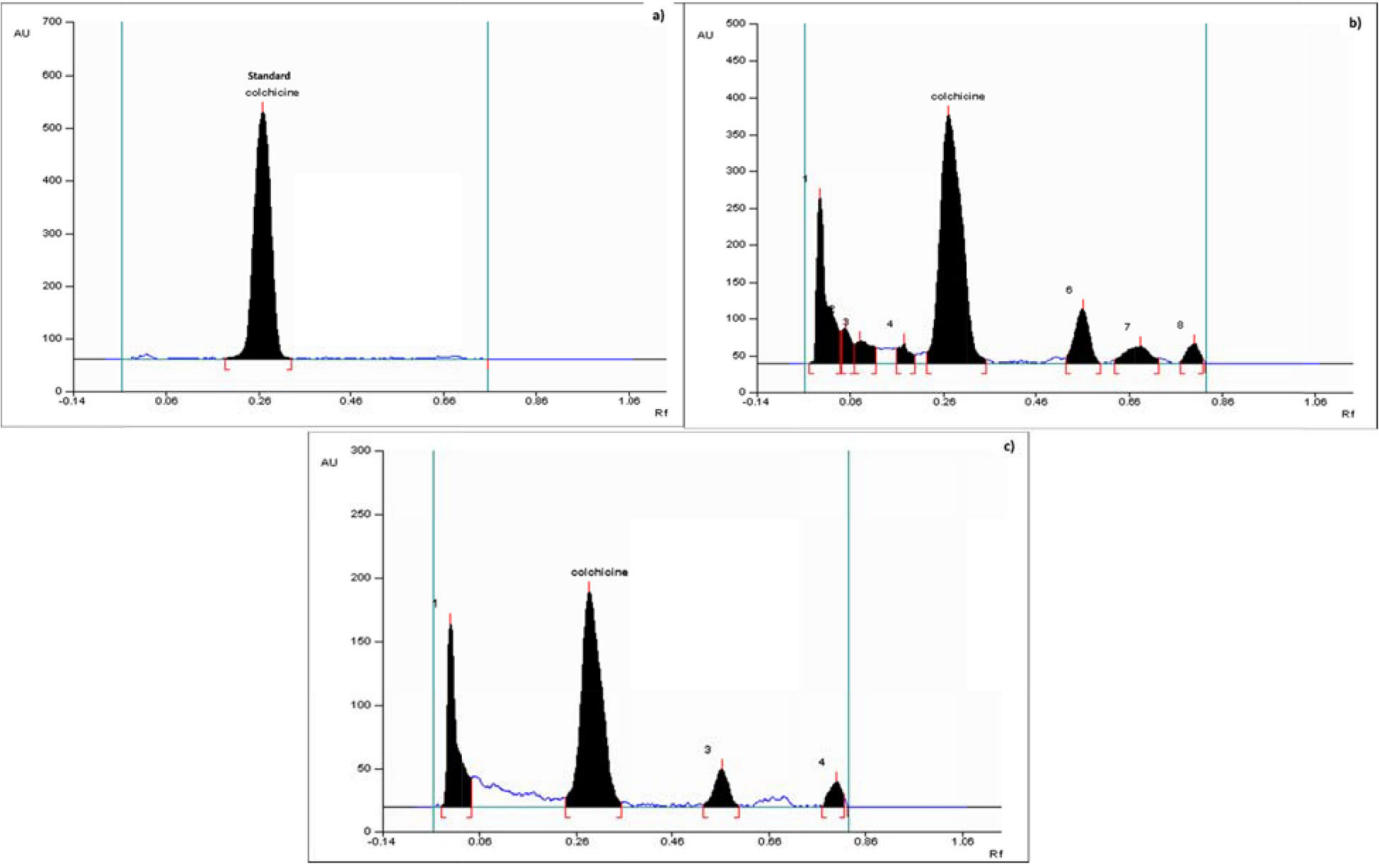
**a** Densitogram obtained from standard colchicine, **b** and **c** Densitograms obtained from different populations of *G. superba*collectedfromdarjeeling (West Bengal) and Gangtok (Sikkim)

**Fig. 5:**
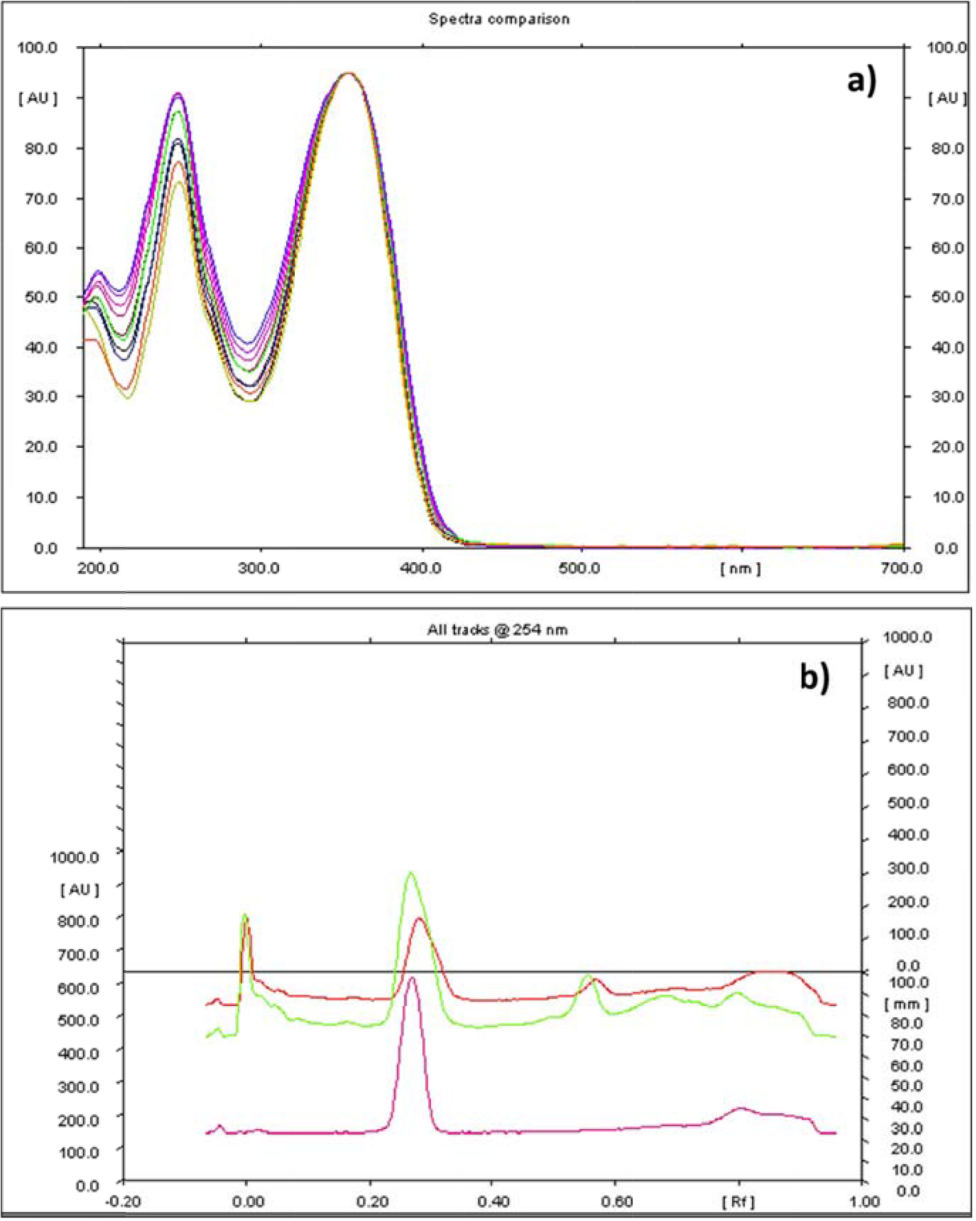
**a** Spectrum of colchicine overlaid with the corresponding peak from different populations; **b** HPTLC (overlay) of different populations of *G. superba* with standard colchicine

Preparatory TLC investigations certified the use of toluene-methanol solvent system [85:15 (v/v)] as ideal mobile phase that resulted in a clear band for colchicine at 0.27 R_f_ value besides generating spots that were well-resolved for the analysed samples (Fig. 4). The spots were recorded at 254 nm following methanolic H_2_SO_4_ [10% (v/v) spraying]. Preliminary HPTLC fingerprinting was conducted on the standard compound and optimization of the parameters was performed. Under similar parameters, the patterns of fingerprinting for the samples were documented. At R_f_ 0. 27, colchicine was recorded to be present. The patterns of the 3-D densitogram of the standard and the samples showed superimposable peak corresponding to the R_f_ value of 0.27. Exact matching of the characteristics spectrum in relation to this peak was also noticed, specifying the compounds in correspondence with the R_f_ value of 0.27 for the test samples and the standard being identical. The colchicine spectrum was found to overlay with the corresponding peak from plant samples that is presented in Fig. 5a. Peak purity was verified by matching the absorption spectra of the test samples and the standard; intelligible superimposibility reflected the peak purity (Fig. 5b). The calibration curve linearity was achieved between 1000-5000 ng in which the correlation coefficient was reported to be 0.97. The regression equation for the calibration plot was Y = 9178 + 4.527*X. The linear regression results are presented in Table 4.

**Table 4:** Method validation parameters for the quantification of colchicine

Sr.no.	Parameters	Colchicine
1	Instrument precision [%CV, *n*=10]	0.44
2	Repeatability [%CV, *n*=10]	0.58
3	Linearity range (ng/spot; *n*=12^a^	1000–5000
4	Correlation coefficient (*r*^*2*^)	0.975
5	Regression equation (Linear)	Y= 9178+ 4.527*X
6	Calculated SD value (CATS software)	10.27
7	^b^Limit of detection (LOD) (ng) [3 x SD/S] (ng/band)	70
8	^b^Limit of quantitation (LOQ) (ng) [10 x SD/S] (ng/band)	210
9	R_f_ and λmax	0.27; 254 nm

^a^Four concentration levels in triplicate; ^b^SD is the standard deviation of the blank response and S is the slope of the calibration plot

#### Method validation

Validation of the present HPTLC methodology was carried out for accuracy, repeatability and precision (Table 4). The present method was found to be specific for colchicine since it resolved the compound (R_f_ = 0.27) well in the existence of other phytochemicals in *G. superba* (Fig. 4). The relationship between response *i.e.,* peak area and colchicine amount ranging from 1.0 to 5.0 μg per band was found to be linear and the correlation coefficient was found to be 0.97 (Table 4). Same spot of colchicine was scanned ten times (%CV = 0.44) in order to study instrument precision.

Eleven applications of the identical standard solution (% CV = 0.58) was used to find out the repeatability of the method. By addition of known colchicine amounts to tuber extract, the method accuracy was evaluated at three different levels *viz.* 50, 100, and 150%. Recovery at the three levels was noted as 100.17%, 99.87%, and 100.2%, respectively (Table 5). Precision and repeatability were determined by the assessment of intra-day as well as interday variations which were observed to be insignificant. The % RSD was found to be 0.37 % and 0.51% respectively (Tables 5). The method specificity was evaluated by furnishing the standard colchicine spectrum and the peak in correspondence associated with the samples in a range of 400-800 nm. The spectra (Fig. 5) derived from the pure marker compound and the marker present in tuber powder were matching precisely, exhibiting no influence of other phytochemicals. The LOD and LOQ were determined via employing the equations; LOD = 3XN/B; LOQ = 10XN/B (N= standard deviation of the peak area of the standard; *n*=3, considered as a measure of the noise; B= slope of the corresponding calibration plot). The LOD and LOQ were found to be 70 ng per band and 210 ng per band, respectively.

**Table 5:** Precision and recoveries of colchicine from plant samples

Sr. no.	Parameters	Colchicine
	**Precision and accuracy**	
1	Intra-day RSD (%), *n*=5	0.37
2	Inter-day RSD (%),*n*=5 (day-1/day-2/day-3)	0.37/0.49/0.51
	**Recovery**	
3	Amount of standard in GS samples (μg mg^−1^) containing highest cochicine	7.57
4	Amount of standards added in GS sample (μg mg^− 1^)	4/8/12
5	Amount of standard found (μg mg^−1^)	11.59/15.55/19.61
6	Recovery (%)	100.17 /99.87/100.2

### Quantification of colchicine in *G. superba* chemotypes

**Fig. 6:**
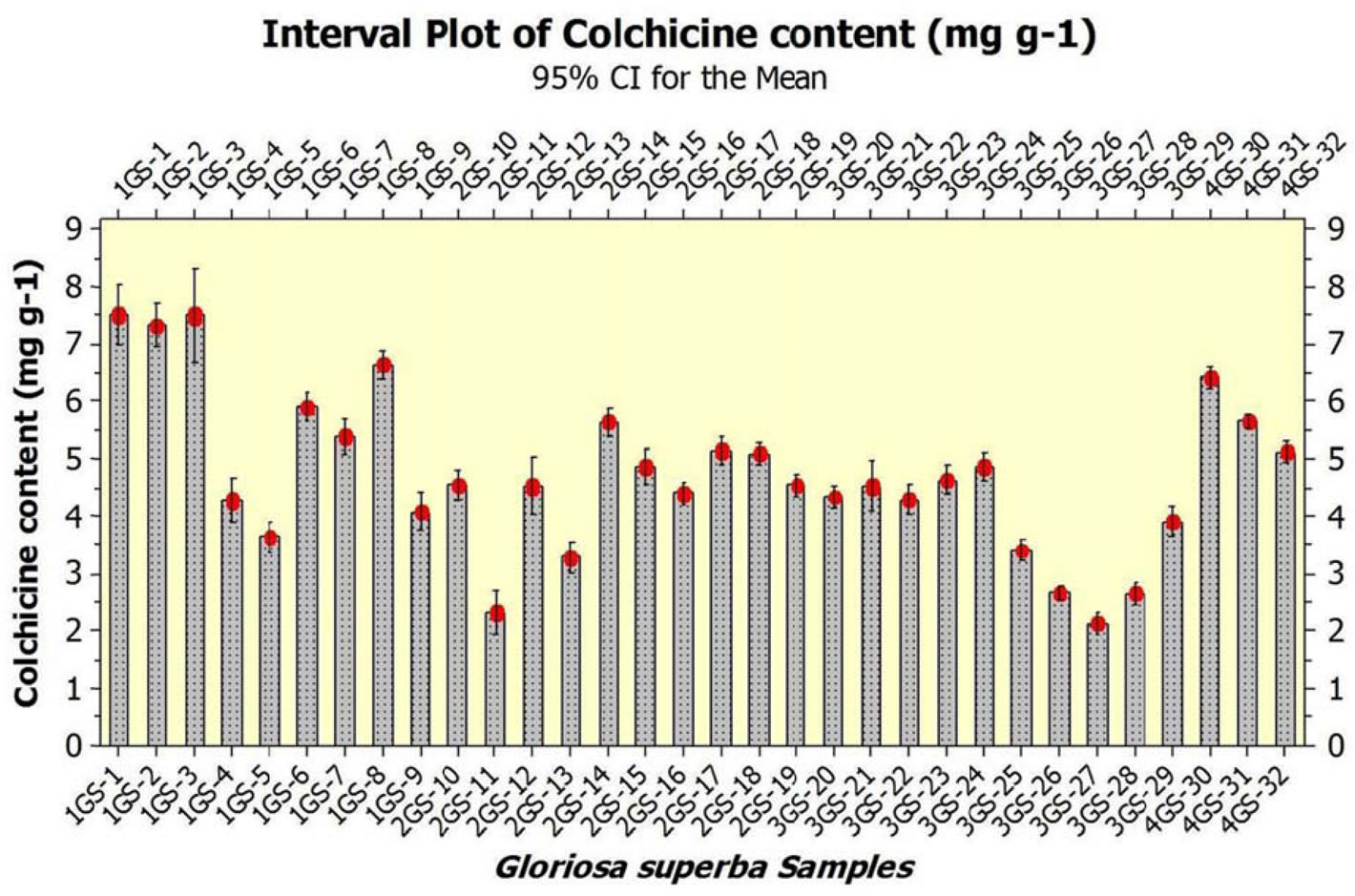
A comparative account on colchicine content among 32 *G. superba* populations following HPTLC quantification

**Fig. 7:**
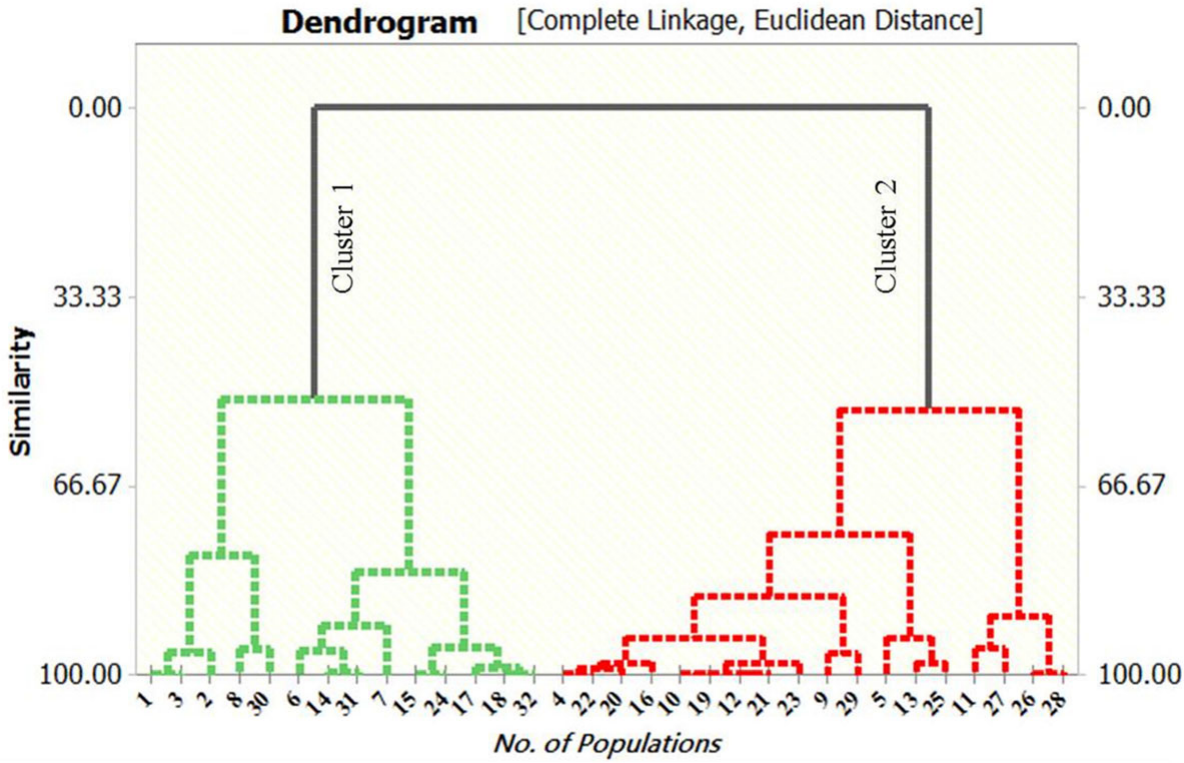
Dendrogram based on colchicine content of *G. superba* collected from different natural habitats in India where cluster 1 represents elite chemotypes and cluster 2 represents other plant samples

The present method was used to explore the colchicine amount in the tubers of *G. superba* chemotypes. The peak area parameter was used to determine the content (mg g^-1^) of the bioactive marker and the data are presented in Table 6. Colchicine content ranged between 2.12 - 7.58 mg g^-1^ among 32 *G. superba* populations collected from 32 different districts across 11 states of India*.* Total three *G. superba* chemotypes *viz*. GS- 3 (collected from Kalimpong, West Bengal) (7.58 mg g^-1^), GS- 1 (collected from Darjeeling, West Bengal) (7.57 mg g^-1^), and GS- 2 (Gangtok, Sikkim) (7.37 mg g^-1^) were screened as elite germplasms. Chemotypes: GS- 27 (2.12mg g^-1^), GS- 28 (2.67 mg g^-1^), GS- 26 (2.68 mg g^-1^), and GS- 11 (2.37 mg g^-1^) collected from different locations of Punjab and Jharkhand, respectively exhibited least amount of colchicine. The results indicate a clear difference in the colchicine amounts in 32 different populations collected from 32 different geographical locations of India **(**Table 6**)**. The populations collected from Eastern Himalayan region at higher altitudes possessed significantly elevated amount of colchicine than that of Western Himalayan regions and Indo - Gangetic plains. Figure 6 depicts the comparative account on colchicine content among 32 *G. superba* populations following HPTLC quantification. UPGMA hierarchical dendrogram was performed on the basis of colchicine content in which fourteen samples *viz*. GS- 1, GS- 2, GS- 3, GS- 6, GS- 7, GS- 8, GS- 14, GS- 15, GS- 17, GS- 18, GS- 24, GS- 30, GS- 31 and GS- 32 were grouped together in one cluster and rest of the eighteen samples formed the second cluster. Figure 7 presents a dendrogram based on colchicine content in *G. superba* collected from different natural habitats across India where cluster 1 represents the elite chemotypes and cluster 2 represents other plant samples. Variations in colchicine content were attributed to altitudinal variation which may be implicated to many other factors such as temperature, UV-radiation, edaphic factors and many more. Polyploidy in the mountain populations of *G. superba* may also play a crucial role in determining the phytochemical content in the said chemotypes. The present study provides important chemical footprint in the domain of bioactive compound (colchicine) mining in *G. superba*.

**Table 6:** Colchicine content in the *G. superba* collected from 32 different populations in India

Samples	Region, state and population type	Colchicine content (mg g^**−1**^)
GS −1	Darjeeling (West Bengal) (wild) (West Bengal)	7.57 ± 0.48
GS −2	Gangtok (Sikkim) (wild)	7.37 ± 0.26
GS −3	Kalimpong (West Bengal)	7.58 ± 0.63
GS −4	Siliguri (West Bengal)	4.32 ± 0.30
GS −5	Kolkata (West Bengal) (wild)	3.67 ± 0.27
GS −6	Bhubaneswar (Orissa) (wild)	5.90 ± 0.24
GS −7	Koraput (Orissa) (wild)	5.43 ± 0.21
GS-8	Chilika (Orissa) (wild)	6.68 ± 0.04
GS-9	Puri (Orissa) (wild)	4.12 ± 0.19
GS-10	Konark (Orissa) (wild)	4.57 ± 0.48
GS −11	Ranchi (Jharkhand) (wild)	2.37 ± 0.26
GS −12	Gaya (Bihar) (wild)	4.58 ± 0.63
GS −13	Sitamarhi (Bihar) (wild)	3.32 ± 0.30
GS −14	Sonbhadra (Uttar Pradesh) (wild)	5.67 ± 0.27
GS −15	Chandauli (Uttar Pradesh) (wild)	4.90 ± 0.24
GS −16	Mirzapur (Uttar Pradesh) (wild)	4.43 ± 0.21
GS −17	Varanasi (Uttar Pradesh) (cultivated)	5.18 ± 0.04
GS-18	Lucknow (Uttar Pradesh) (cultivated)	5.12 ± 0.19
GS-19	Sitapur (Uttar Pradesh) (wild)	4.57 ± 0.48
GS-20	Dehradun (Uttarakhand) (wild)	4.37 ± 0.26
GS −21	Haridwar (Uttarakhand) (wild)	4.58 ± 0.63
GS −22	Pantnagar (Uttarakhand) (wild)	4.32 ± 0.30
GS −23	Hisar (Haryana) (wild)	4.67 ± 0.27
GS −24	Kurukshetra (Haryana) (wild)	4.90 ± 0.24
GS −25	Chandigarh (wild)	3.43 ± 0.21
GS −26	Patiala (Punjab) (wild)	2.68 ± 0.04
GS −27	Phagwara (Punjab) (wild)	2.12 ± 0.19
GS-28	Ludhiana (Punjab) (wild)	2.67 ± 0.27
GS-29	Amritsar (Punjab) (wild)	3.90 ± 0.24
GS-30	Palampur (Himachal Pradesh) (wild)	6.43 ± 0.21
GS-31	Solan (Himachal Pradesh) (wild)	5.68 ± 0.04
GS-32	Jammu (Jammu and Kashmir) (wild)	5.12 ± 0.19

Results (% of dry weight) are given as mean value ± standard deviation (*N* = 3)

The values followed by the same letter are not significantly different using Tukey test (*P* ≤ 0.05)

## Discussion

### Screening and optimization of significant extraction parameters employing RSM

Present study was conducted for the optimization of non-conventional extraction method (MAE) by using Plackett-Burman and Box-Behnken design (BBD)*.* Plackett-Burman design was employed for the screening of seven independent variables, and only four parameters *viz*. power of microwave, time of irradiation, solvent composition and pH exhibited remarkable impact on colchicine yield. BBD was used to evaluate the linear, square and interactive effects of these four MAE independent variables in order to achieve maximum yield of colchicine from *G. superba*. At these optimized microwave parameters, maximum predicted yield achieved for colchicine was found to be 7.51 mg g^-1^. Another study reported the effect of three extraction parameters (interaction of time, power and methanol concentration) on extraction of colchicine with BBD [45]. However, the conventional methods are expensive as they consume larger amount of solvents, take more time for extraction and are not eco-friendly compared to the green methods. In this present investigation, MAE exhibited better yield of colchicine consuming less time (6.4 min) and 30% aqueous ethanol solvent. Similar results on the green extraction were obtained using MAE and non-toxic solvents in various bioactive compounds *viz.* pentacyclic triterpenoids, seco-iridoids, xanthone glycosides etc. [35, 41, 43].

### Quantification of colchicine in *G. superba* chemotypes

The chemotaxonomic study is essential to identify the elite germplasms of *G. superba* for the commercial exploration of targeted metabolite. In present study, validated HPTLC methodology was developed and used to screen the high metabolite yielding chemotypes in *G. superba*. Due to excessive exploitation and indiscriminate collection, the Glory lily population is on the verge of extinction from the wild in India. Therefore, elite chemotype(s) identified in present study will serve as a source of good quality planting material (QPM) for commercial cultivation and will meet the ever-expanding demand of the herbal pharmaceutical industry.

Earlier, colchicine content was compared in two genera of the family Colchicaceae viz. *Gloriosa* and *Colchicum*. The results indicated higher colchicine accumulation in *Gloriosa* (0.9%) in comparison to *Colchicum* (0.62%) [46]. *G. superba* is indigenous to the subcontinent and grows in regions with diverse altitudinal zones (up to 2500 m). Therefore, various populations of the plant growing in different climate show variation in their metabolic profiling. Hence, such populations can be termed as chemotypes. Quantitative analysis of colchicine among 32 *G. superba* populations growing in an altitudinal range of 0–2045 m revealed that altitude plays a crucial role in colchicine content among chemotypes. Similar intra-specific modulation in the quantity of various metabolites along the altitudinal gradient has been noted earlier in diverse plant species [47, 48]. Such altitude-influenced variation in secondary metabolite profiling was attributed to several physiological, ecological and environmental factors viz. elevated UV-B radiation, decreased temperatures and radical scavenging properties of phenolics [47, 49–56]. Misra et al. [31] also developed a HPTLC method for colchicine and gloriosine quantification in *G. superba* from Sikkim Himalayas. However, their study was limited to only five germplasms of *G. superba*. In addition, another previous study assessing the karyomorphology of *G. superba* collected from Siliguri, Darjeeling and Sikkim revealed that the Sikkim plants exhibited tetra ploidy while the others were diploid [57]. Therefore, higher colchicine content in uphill populations of *G. superba* may also be implicated to the ploidy levels of the collected samples. However, further conclusive study is needed to prove this hypothesis. In some earlier studies, natural or induced polyploidization has been correlated to the modulated quality and quantity of secondary metabolites in different medicinal plants [58, 59]. It is well established that species growing in different altitudes show diverse biological characters varying with altitude, mainly attributed to their adaptation to different environmental conditions. In the present investigation, a significant increase in the amount of colchicine in high altitude populations may be a result of adaptation to different temperature, exposure to UV-radiation, humidity, soil types and aridity.

## Conclusion

In this present study, an optimized and validated method for simultaneous MAE and HPTLC-quantification of anticancer colchicine in Indian natural populations of *G. superba* was developed for identification of elite chemotypes. All statistical indications in this study support RSM as a successful tool to optimize MAE from *G. superba* tubers in which only four parameters such as power of microwave, time of irradiation, solvent composition and pH had exhibited noteworthy influence on colchicine yield as recorded by their *P* values at the level of 5% (*P* < 0.05). The results of HPTLC study suggest that the validated method is linear, repeatable, selective, and accurate within established ranges. This developed method demonstrated prompt as well as efficient analytical technique for screening of large number of samples over short period of time. Colchicine content ranged between 2.12–7.58 mg g^− 1^ among 32 *G. superba* populations collected from 32 different districts across 11 states of India*.* Maximum amount of colchicine content was reported from the samples collected from the Eastern region of India: Darjeeling- West Bengal (7.58 mg g^− 1^) and Gangtok-Sikkim (7.37 mg g^− 1^) and thus screened as elite germplasms among different populations. These elite *G. superba* populations in terms of phytochemical content can be conserved and mass propagated by the local people. Variations in colchicine content were attributed to altitude which may be implicated to many other factors such as temperature, UV-radiation, edaphic factors etc. Polyploidy in the mountain populations of *G. superba* may also play a crucial role in determining the phytochemical content in the said chemotypes. The present study provides a valuable phytochemical profiling for different *G. superba* plant accessions which will be beneficial for pharmaceutical industries.

## Methods

### Plant material, reagents and standard solutions

Small parts of tubers from 32 populations of *G. superba* belonging to identical growth stage (flowering stage) were collected from diverse agroclimatic zones of India, from the sea level to 2045 m altitude during the year 2018-2019 (Table 7). The plants were collected from different places after taking permission from the all the landowners allowing us to sample on their private land. The plant material was identified as *G. superba* by Dr. R.C. Gupta, Professor, Department of Botany, Punjabi University, Patiala, Punjab. The plant material (voucher specimen no 11122018) has been stored in the Department of Biotechnology, Lovely Professional University, Phagwara, Punjab.

**Table 7:** Geographical locations of *G. superba* collected from different natural habitats in India

Samples	Region, state and population type	Coordinates	Altitude (m) above sea level (asl)
GS −1	Darjeeling (West Bengal) (wild) (West Bengal)	27.0500° N, 88.2667° E	2045
GS −2	Gangtok (Sikkim) (wild)	27.3300° N, 88.6200° E	1650
GS −3	Kalimpong (West Bengal)	27.0600°N 88.4700°E	1250
GS −4	Siliguri (West Bengal)	26.7100° N, 88.4300° E	122
GS −5	Kolkata (West Bengal) (wild)	22.5726°N 88.3639°E	11
GS −6	Bhubaneswar (Orissa) (wild)	20.2700° N, 85.8400° E	45
GS −7	Koraput (Orissa) (wild)	18.8083° N, 82.7083° E	649
GS-8	Chilika (Orissa) (wild)	19°4300 N 85°1900 E	1
GS-9	Puri (Orissa) (wild)	19°48′38″N 85°49′53″E	0.1
GS-10	Konark (Orissa) (wild)	19°53′27″N 86°06′01″E	2
GS −11	Ranchi (Jharkhand) (wild)	23.3600°N 85.3300°E	651
GS −12	Gaya (Bihar) (wild)	24.7500°N 85.0100°E	111
GS −13	Sitamarhi (Bihar) (wild)	26.6°N 85.48°E	70
GS −14	Sonbhadra (Uttar Pradesh) (wild)	24°41′23″N 83°3′55″E	80
GS −15	Chandauli (Uttar Pradesh) (wild)	25.27°N 83.27°E	70
GS −16	Mirzapur (Uttar Pradesh) (wild)	25.146°N 82.569°E	80
GS −17	Varanasi (Uttar Pradesh) (cultivated)	25.2800° N, 82.9600° E	81
GS-18	Lucknow (Uttar Pradesh) (cultivated)	26.8000° N, 80.9000° E	123
GS-19	Sitapur (Uttar Pradesh) (wild)	27.57°N 80.66°E	138
GS-20	Dehradun (Uttarakhand) (wild)	30.3180° N, 78.0290° E	636
GS −21	Haridwar (Uttarakhand) (wild)	29.945°N 78.163°E	314
GS −22	Pantnagar (Uttarakhand) (wild)	28.97°N 79.41°E	234
GS −23	Hisar (Haryana) (wild)	29°09′N 75°42′E	215
GS −24	Kurukshetra (Haryana) (wild)	29.965717°N 76.837006°E	260
GS −25	Chandigarh (wild)	30°45′N 76°47′E	321
GS −26	Patiala (Punjab) (wild)	30.34°N 76.38°E	350
GS −27	Phagwara (Punjab) (wild)	31.22°N 75.77°E	234
GS-28	Ludhiana (Punjab) (wild)	30.91°N 75.85°E	262
GS-29	Amritsar (Punjab) (wild)	31.64°N 74.86°E	256
GS-30	Palampur (Himachal Pradesh) (wild)	32.109722°N 76.536641°E	1700
GS-31	Solan (Himachal Pradesh) (wild)	30.905°N 77.097°E	1600
GS-32	Jammu (Jammu and Kashmir) (wild)	32.73°N 74.87°E	350

The tubers were air-dried at 25-32 °C and following their segmentation into small fragments to accelerate drying. In order to prepare a homogeneous powder for analysis, the dried tubers were ground to powder and the powder was passed through a sieve. The obtained powder was stored in dark at room temperature for further analysis.

HPLC grade solvents were procured from E. Merck (Mumbai, India). MilliQ PLUS purification system (Millipore, USA) was used to obtain ultra-pure water. Standard colchicine was procured from Sigma-Aldrich, USA. All analytical grade chemicals were used in the assays. Colchicine (10 mg) was dissolved in 10 ml methanol to prepare a stock solution (1 mg/ ml). To prepare the calibration curve, the stock was serially diluted to produce standard solutions of various concentrations.

### Sample preparation

In Preliminary experimentation, one gram of shade-dried powdered material from each population of *G. superba* was extracted individually with methanol (3x25 ml). The individual flasks were positioned on a gyratory shaker at 100 rpm for 6 h. After leaving the suspension at room temperature (22±2 °C) overnight, pooling of all extracts was carried out.

In addition, different notable extraction parameters were optimized to evaluate the effect of MAE. In this, the powdered tuber (0.5 g each) of *G. superba* was extracted with MAE employing a microwave oven (model: LG MC7849HS) in 100 mL closed vessel units by employing different extraction parameters. Filter paper (Whatman No1) was used to filter the extracts following their evaporation in vacuum employing a rotary evaporator (model: Eyela N-1100, China) to generate a solid mass of the extract which was then poured in methanol (1 ml) and then it was sonicated (10 min). Chromatography and colchicine quantification were performed with this sample.

### Experimental design for MAE optimization by response surface methodology (RSM)

Experimental designing was performed by Minitab statistical software. A two-stage experimental procedure was adopted: i) to test the significant independent parameters, Plackett–Burman design was used and ii) in order to verify the optimum level and probable interactions among important parameters, composite design was employed.

#### Plackett-Burman model

In order to optimize extraction of *G. superba* tuber-derived colchicine*,* Plackett Burman model was employed for analysing the significant parameters.

Plackett–Burman design is based upon the following first-order model:

2 $$ \mathrm{Y}={\beta}_0+\sum {\beta}_{\mathrm{i}}{\mathrm{X}}_{\mathrm{i}} $$

(where Y is expected target function, β_0_ is scaling constant and β_i_ denotes regression coefficients)

The influence of different variables *viz.* power of microwave (300 and 600 W), time of irradiation (3 and 9 min), particle size (0.5 and 1.0 mm), composition of solvent (20%-40% aqueous ethanol), solid-solvent ratio (15 and 30 g/ml), pH (2 and 6) and extraction steps (1 and 2) on colchicine extraction were investigated. Two level experiment was carried out where (+) depicts maximum and (−) depicts minimum values respectively (Table 1).

Twelve experiments were conducted in duplicates to test all the above-mentioned variables (Table 1). The significant parameters were investigated by the analyses of regression at 5% level (*P* < 0.05) as exhibited in Table 2.

#### Box-Behnken Design (BBD)

During the experiments, 3 levels (-1, 0, +1) model of BBD with 4 factors were employed to evaluate the important parameters of MAE. Three noteworthy factors for microwave extraction such as power of microwave (A: 300-600 W), time of irradiation (B: 3-9 min), solvent composition (C: 20%-40% aqueous ethanol) and pH (2-6) were employed as independent variables where 27 experimental runs were carried out during the experiments (Table 3). To evaluate the stability of microwave strategy and to achieve optimum colchicine yield (%), both higher as well as lower response values were employed. BBD was applied for colchicine yield optimization from *G. superba*. The RSM model response value optimization of colchicine from *G. superba* is performed on the basis of the following quadratic equation:

3 $$ \mathrm{Y}={\beta}_0+\sum \limits^n\left({\beta}_i{X}_i\right)+\sum \limits^n\left({\beta}_{ii}{X_i}^2\right)+\sum \limits^n\left({\beta}_{ij}{X}_i{X}_j\right) $$

[where Y is desired bioactive chemical’s predicted response value, n is total number of independent variables with n=3, *β*_0_, *β*_*i*_, *β*_*ii*_, *and β*_*ij*_ are intercept, linear, quadratic and interaction effects’ regression coefficients respectively; *X*_*i*_ and *X*_*j*_.are various extraction parameters (i and j as 1 to n variables)]. Influence of MAE application on colchicine yield was analysed for significant variances (*p*<0.01) (Table 3).

### HPTLC analysis for colchicine quantification

The HPTLC machinery was composed three components *viz.* CAMAG Linomat 5 automatic sample-applicator (Muttenz, Switzerland), CAMAG TLC scanner 3 and an attached software (CATS; version: 1.4.4.6337). TLC plates of precoated silica gel 60 F_254_ (Merck KGaA 1.05554. 0007; 20 cm x 10 cm in dimension and 0.25 mm in layer thickness) were used as the stationary phase. Samples were put in to the plates as 6 mm wide bands, with 10 mm track distance, employing the nitrogen flow equipped sample applicator provided with a 100 μl Hamilton syringe which had a defined 150 nl/s delivery rate. The mobile phase was composed of toluene-methanol 85:15 (v/v) to achieve a development of linear ascending mode up till an extent of 80 mm inside a mobile phase vapor saturated twin trough glass chamber (CAMAG; 20 cm x 10cm) at 22±2 °C and 50% relative humidity for 20 min. After the development, the plates were dried for 10 min at 100 and were derivatized with methanolic sulphuric acid solution [100 ml of 10% (v/v)]. Densitometric scanning was conducted at 254 nm. The slit dimension and the scanning speed were measured at 5 mm x 0.45 mm and 100 nm/s, respectively.

In order to analyse the linearity and calibration, marker stock solutions (1.0, 2.0, 3.0, 4.0 and 5 μl) were employed to the plate to provide quantities from 100 ng to 500 ng per band. Corresponding concentrations were employed to plot the peak areas and in order to produce the calibration equation, regression analysis was performed. To analyse the underground tuber powder extract, 10 μl of the obtained sample as described in section 2.2 was administered to the plate. Accepting the marker purity as 100%, following the development, derivatization, scanning and peak area measurement, the content of colchicine was calculated.

Extracts from the plant samples and the standard solutions (1.0, 2.0, 3.0, 4.0 and 5.0 μl) were administered to TLC plates and were analysed by the previously described method. Following resolving the peak areas, a calibration plot was generated via peak area plotting against the standard quantity of colchicine employed. This calibration plot was applied to calculate the amounts of colchicine present in the chemotypes.

### Method validation

The method validation was determined via analysing the peak purity, linearity, the limit of detection (LOD), repeatability (Table 4), percentage recovery, intra-day as well as intermediate precision (Table 5) for colchicine obtained from tubers. Each of the standard solutions of colchicine (in equivalence of 100, 200, 300, 400 and 500 ng/ band) was administered in triplicate. Against the respective colchicine amount, the peak area plotting was carried out to find the calibration plot and the linearity range was also decided. Six times scanning was performed to scan the same band for colchicine (500 ng) to check the precision of the instrument. For peak area and R_f_, the mean and standard deviation with the percentage of coefficient of variation were determined. The results acquired from the investigations spotting instrument precision or repeatability is presented in Table 4. Standard solution was administered to the plate (in triplicate) and the % CV was determined to test the repeatability evaluating the colchicine band. 50, 100 and 150 % colchicine to the sample were added to test the method accuracy via ascertaining recovery at three levels. To 1.0 g powdered plant material containing around 0.76 mg colchicine, standard colchicine of known amounts was added, the sample was extracted and the colchicine amounts were decided following the above-described method. Recovery was determined for all three levels (Table 5). Precision was calculated via analysing the sample solution in three bands per plate provided on three plates to determine the intra-day precision. To determine the intermediate precision, sample solution in three bands per plate was analysed on day two and % CV was also enumerated (Table 4). The test samples’ corresponding peak (in 200-800 nm range) in accordance to the standard colchicine’s absorbance spectrum was employed in determining the method specificity. Standard solutions in different dilutions were employed using methanol (blank) to resolve the limit of detection and limit of quantification (LOD and LOQ) respectively.

## Data Availability

The datasets used and/or analysed during the current study available from the corresponding author on reasonable request.
